# Glucose recovery from aqueous solutions by adsorption in metal–organic framework MIL-101: a molecular simulation study

**DOI:** 10.1038/srep12821

**Published:** 2015-08-05

**Authors:** Krishna M. Gupta, Kang Zhang, Jianwen Jiang

**Affiliations:** 1 Department of Chemical and Biomolecular Engineering, National University of Singapore, 117576, Singapore

## Abstract

A molecular simulation study is reported on glucose recovery from aqueous solutions by adsorption in metal-organic framework MIL-101. The F atom of MIL-101 is identified to be the most favorable adsorption site. Among three MIL-101-X (X = H, NH_2_ or CH_3_), the parent MIL-101 exhibits the highest adsorption capacity and recovery efficacy. Upon functionalization by -NH_2_ or -CH_3_ group, the steric hindrance in MIL-101 increases; consequently, the interactions between glucose and framework become less attractive, thus reducing the capacity and mobility of glucose. The presence of ionic liquid, 1-ethyl-3-methyl-imidazolium acetate, as an impurity reduces the strength of hydrogen-bonding between glucose and MIL-101, and leads to lower capacity and mobility. Upon adding anti-solvent (ethanol or acetone), a similar adverse effect is observed. The simulation study provides useful structural and dynamic properties of glucose in MIL-101, and it suggests that MIL-101 might be a potential candidate for glucose recovery.

The U.S. Energy Information Administration predicted that the global energy demand would rise substantially by 45% from 2010 to 2035^1^. To meet the rapid growth of energy demand and meanwhile to tackle the increasing environmental pollution, there has been considerable interest in renewable and environmentally benign energy resources. In particular, largely available biomass is under extensive studies to be converted into biofuels, chemicals and biomaterials^2^,^3^. Among different types of biomass, cellulose is a major component with a global quantity of 700,000 billion tons; however, only 0.1 billion tons of cellulose is currently being used for the production of paper, textiles, etc^4^. Therefore, great potential exists to explore the untapped cellulose.

Before converted into valuable products, cellulose is required to be dissolved and hydrolyzed. As a polysaccharide, cellulose is composed of linear chains with β (1 → 4) D-glucose units (i.e. glucosidic linkage). These chains are hydrogen-bonded (H-bonded) leading to strong structural strength and thus cellulose cannot be readily dissolved by common organic/inorganic solvents. In this context, ionic liquids (ILs) have turned out to be intriguing solvents to dissolve cellulose^5^. After dissolution, cellulose is enzymatically hydrolyzed to yield glucose (or other oligosaccharides). Glucose is a useful precursor for biofuels, pharmaceuticals and chemicals^6^, and thus required to be recovered from aqueous solutions. In certain case, the solutions may contain IL because the hydrolysis is usually conducted in the presence of IL to minimize inhibitory effect on enzyme^7^,^8^,^9^.

Among a handful of techniques, adsorption in porous materials is energy-efficient and cost-effective for biomass processing^10^. Various materials such as mineral surfaces, ion-exchange resins and zeolites have been tested for glucose recovery through experimental or theoretical approaches. For example, hematite and quartz were used for the adsorption of glucose and other bio-products^11^. Ion-exchange resins were examined for the recovery of different carbohydrates including glucose, sucrose and fructose from aqueous solutions^12^,^13^,^14^. In Na^+^, Mg^2+^, Ca^2+^ and Al^3+^ exchanged zeolite-Y, the amount of glucose adsorbed was found to increase in the order of NH_4_-Y < Mg-Y < Ca-Y < Na-Y^15^. Similarly, cation-exchanged zeolite-X were also investigated for the adsorption of glucose and sugar, the amount of adsorption increased as Ba-X < Mg-X < Ca-X < K-X < Sr-X < Na-X^16^. Different types of zeolites were tested for glucose recovery from aqueous solutions as well as water/1-ethyl-3-methyl-imidazolium acetate [C_2_mim][Ac] mixture^17^. On the other hand, the adsorption of glucose in hydrophobic zeolites was simulated and the contributions of enthalpy, entropy and free energy to the transfer of glucose from aqueous phase to zeolite were quantitatively analyzed^18^.

In the above discussed studies, the adsorption amount of glucose usually is not sufficiently high. An ideal adsorbent should possess a large surface area and pore volume, thus leading to high adsorption performance. Emerged as a new class of porous materials, metal-organic frameworks (MOFs) have received tremendous interest. They can be synthesized by the judicious selection of inorganic and organic building blocks, and their surface area and pore volume can be readily tuned with a wide variety of topologies and dimensions. Consequently, MOFs have been considered versatile materials for storage, separation, catalysis, etc^19^. Nevertheless, most current studies for MOFs have been largely focused on the storage of low-carbon footprint energy carriers (e.g. H_2_ and CH_4_) and the separation of CO_2_-containing gas mixtures^20^,^21^,^22^.

In this study, we report a molecular simulation study for glucose recovery by adsorption in a MOF namely MIL-101 (MIL: *Matériaux Institut Lavoisier*). With chromium terephthalate-based mesoporous structure, MIL-101 has a large surface area (3780 m^2^/g) and free volume (1.74 cm^3^/g). It is assembled by corner-sharing supertetrahedra consisting of octahedral Cr_3_O trimers and 1,4-benzenedicarboxylic acids^23^. Recently, phosphotungstic acid was encapsulated in MIL-101 and examined for the selective dehydration of glucose and fructose^24^. Moreover, sulfonic acid groups decorated MIL-101 was tested for cellulose hydrolysis, and distinct and clean catalytic activity was observed^25^. These remarkable properties have called considerable interest in the use of MIL-101 for biomass processing. To investigate the effect of framework functionality on glucose recovery here, MIL-101 functionalized by –NH_2_ and –CH_3_ groups are examined. Figure 1 illustrates the supertetrahedra in MIL-101, MIL-101-NH_2_ and MIL-101-CH_3_. In addition, the effects of [C_2_mim][Ac], ethanol and acetone are also investigated. Our recent study demonstrated that ethanol and acetone may act as anti-solvents for cellulose regeneration^26^, thus it is instructive to explore their effects on glucose recovery.

## Results and Discussion

### Effect of framework functionality

**Figure 2:**
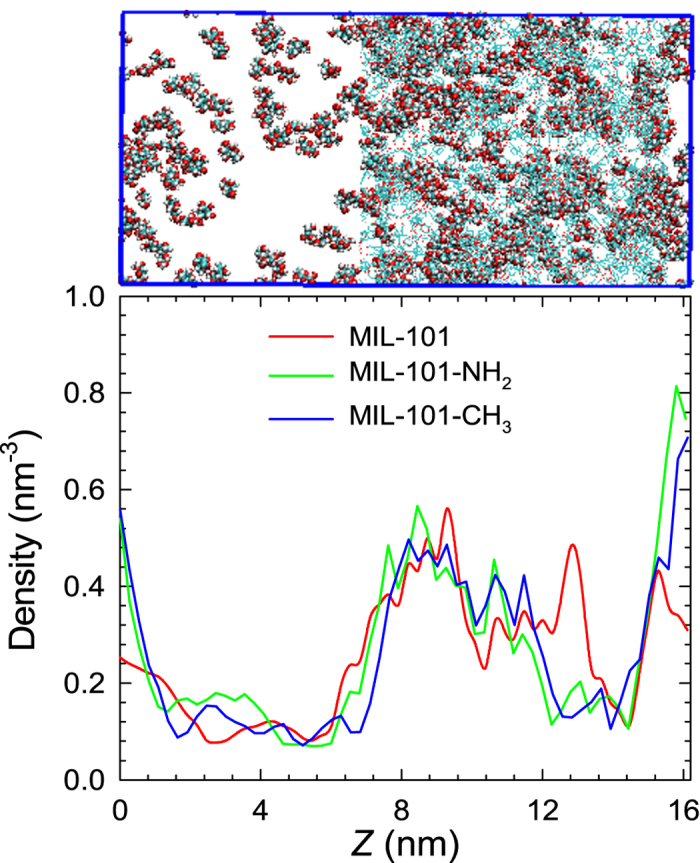
Density profiles of glucose in glucose/water/MIL-101-X systems. The top illustrates a typical simulation snapshot at equilibrium (water molecules not shown).

Upon the initiation of MD simulation, glucose molecules were observed to gradually move from solution to MIL-101-X. Figure S1 of the Supplementary Information shows the numbers of glucose adsorbed in MIL-101, MIL-101-NH_2_ and MIL-101-CH_3_ (systems 1–3) versus simulation time, and similar plots are shown in Figure S2 for systems 4–6 in the presence of IL, ethanol and acetone. Obviously, the numbers remain nearly constant after approximately 20 ns. The adsorption process can be visualized by a movie in the Supplementary Information. Figure 2 shows a typical simulation snapshot at equilibrium and the ensemble averaged density profiles of glucose in glucose/water/MIL-101-X systems. In each system, the maximum density is located between 7 and 16 nm, indicating glucose is adsorbed into the cages in MIL-101-X. Because the cages are not homogeneously distributed, thus the profile is not uniform. Overall, the density in MIL-101 is higher than in MIL-101-NH_2_ and MIL-101-CH_3_. The average numbers of adsorbed glucose molecules are 241.8 in MIL-101, 228.4 in MIL-101-NH_2_ and 228.5 in MIL-101-CH_3_. This reveals that the adsorption capacity of glucose in MIL-101 is reduced upon functionalization, as attributed to the reduced free volume or porosity in the presence of functional groups. Specifically, the porosity is 0.824 in MIL-101, and reduced to 0.795 in MIL-101-NH_2_ and MIL-101-CH_3_^27^. The recovery efficacy of glucose is quantified by separation factor, defined as (*N*_ad_/*V*_ad_)/(*N*_w_/*V*_w_), where *N*_ad_ and *N*_w_ are the numbers of glucose molecules in adsorbed phase and solution, *V*_ad_ and *V*_w_ are the volumes of the two phases. In MIL-101, MIL-101-NH_2_ and MIL-101-CH_3_, the separation factors are 2.15, 1.81 and 1.79, respectively. Apparently, the separation factor drops upon functionalization.

**Figure 3:**
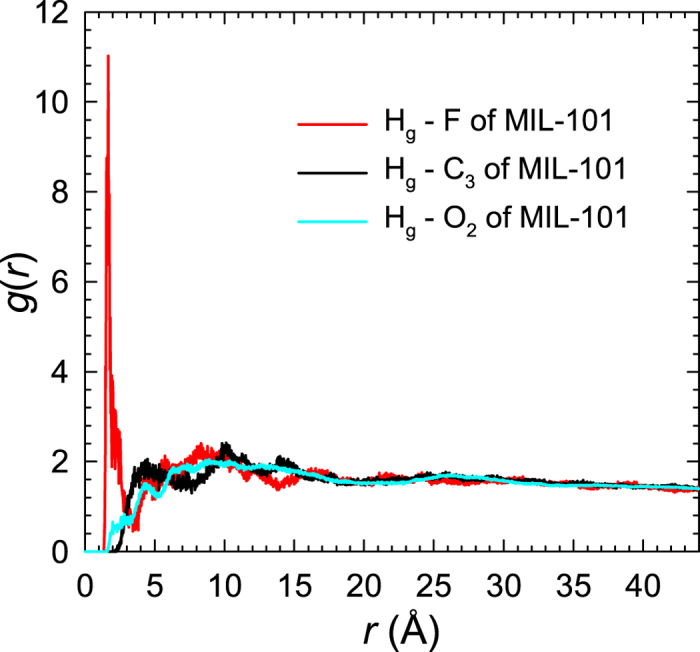
Radial distribution functions between glucose (H_g_ atom) and MIL-101 (F, C_3_, and O_2_ atoms) in glucose/water/MIL-101 system.

The favorable sites for glucose adsorption in MIL-101-X are examined by calculating the radial distribution functions


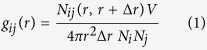


where *r* is the distance between atoms *i* and *j*, 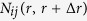 is the number of atom *j* around *i* within a shell from *r* to *r* + Δ*r*, *V* is the system volume, *N*_*i*_ and *N*_*j*_ are the numbers of atoms *i* and *j*, respectively. Figure 3 shows the *g*(*r*) between the H_g_ atom of glucose and the F, C_3_, and O_2_ atoms of MIL-101. No peak is observed around the C_3_ and O_2_ atoms; in contrast, there is a pronounced peak at *r* = 1.8 Å around the F atom. This suggests the F atom, due to its high electronegativity, is the most favorable site for glucose adsorption. Indeed, a hydrogen-bond (H-bond) is formed between the F atom and glucose as evidenced by two geometrical criteria: (1) the distance between a donor and an acceptor ≤ 0.35 nm and (2) the angle of hydrogen-donor-acceptor (between hydrogen-donor and donor-acceptor) ≤ 30° 28,29. In glucose/water/MIL-101 system, the number of H-bonds between all the adsorbed glucose molecules and MIL-101 was estimated to be 58.3.

**Figure 4:**
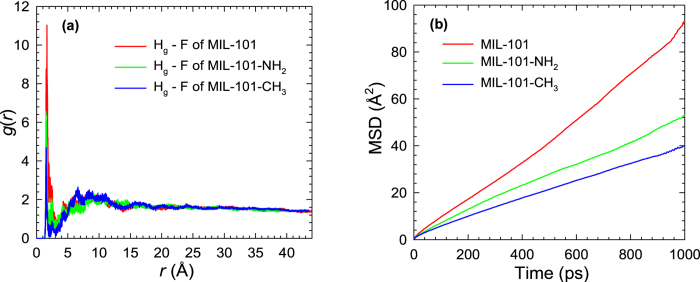
(**a**) Radial distribution functions between the H_g_ atom of glucose and the F atom of MIL-101-X (**b**) Mean-squared displacements of glucose in glucose/water/MIL-101-X systems.

Figure 4a further plots the *g*(*r*) between glucose and the F atom of MIL-101, MIL-101-NH_2_ and MIL-101-CH_3_, respectively. In the three frameworks, the peak appears at nearly the same distance *r* = 1.8 Å. This reflects that the structural arrangement of glucose in the framework is quantitatively identical. Nevertheless, the *g*(*r*) in MIL-101 has the highest peak, followed by those in MIL-101-NH_2_ and MIL-101-CH_3_. The reason is that the F atom is not readily accessible by glucose in the presence of functional group. In other words, the steric hindrance of functional group prevents glucose from being in close contact with the F atom. Furthermore, –CH_3_ is slightly bulkier than –NH_2_ and the steric hindrance is larger, thus the peak in MIL-101-CH_3_ is lower than that in MIL-101-NH_2_.

The mobility of glucose upon adsorption in MIL-101-X is examined by mean-squared displacement (MSD)


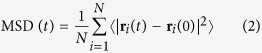


where *N* is the number of glucose and **r**_*i*_(*t*) is the position of the *i*^th^ glucose at time *t*. To improve statistical accuracy, the multiple time-origin method was used to estimate the MSD. Figure 4b shows the MSDs of glucose in glucose/water/MIL-101-X systems. The MSD decreases in the order of MIL-101 > MIL-101-NH_2_ > MIL-101-CH_3_ as attribute to two factors. Firstly, MIL-101 has the largest porosity, thus the mobility in MIL-101 is the highest. While both MIL-101-NH_2_ and MIL-101-CH_3_ possess nearly the same porosity, -CH_3_ group is bulkier than -NH_2_ and thus the mobility is lower in MIL-101-CH_3_. In addition, the MSDs along the z-axis are plotted in Figure S3. Similar trend of these MSDs is observed, i.e., the magnitude of MSD decreases upon functionalization.

### Effects of ionic liquid and anti-solvent

**Figure 5:**
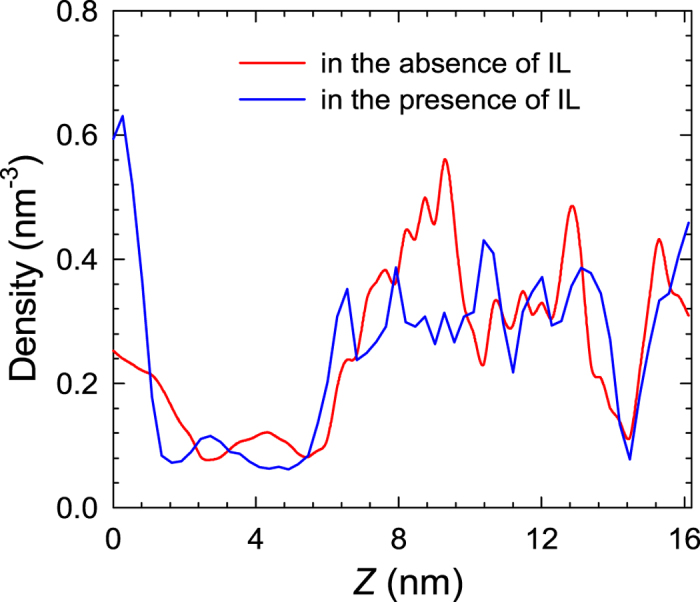
Density profiles of glucose in glucose/water/MIL-101 system in the absence and presence of [C_2_mim][Ac].

As mentioned above, enzymatic hydrolysis of glucose is sometimes performed in the presence of IL. Therefore, IL may exist in the aqueous solution of glucose and it is instructive to evaluate the effect of IL on glucose recovery. Figure 5 shows the density profiles of glucose in glucose/water/MIL-101 system in the absence and presence of [C_2_mim][Ac]. Similar to Fig. 2, the maximum density is located between 7 and 16 nm, implying the adsorption of glucose into the framework. Apparently, the density is higher in the absence of IL. The average number of glucose molecules in MIL-101 in the absence of IL is 241.8, but 216.5 in the presence of IL. This clearly indicates that the adsorption capacity is reduced by the presence of IL. The separation factor also drops from 2.15 to 1.50. As illustrated in Figure S4a, the IL is co-adsorbed into MIL-101 framework thus reducing the adsorption capacity. Figure S4b represents the *g*(*r*) between [C_2_mim]^+^/[Ac]^−^ and the F atom of MIL-101. Due to the electropositive nature, [C_2_mim]^+^ exhibits a pronounced peak. This indicates that the F atom of MIL-101 also acts as adsorption site for IL, and there exists competitive adsorption between glucose and IL. Therefore, the capacity of glucose is reduced in the presence of IL as impurity.

**Figure 6:**
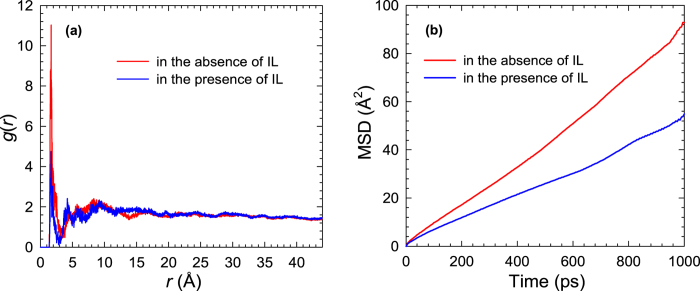
(**a**) Radial distribution functions between the H_g_ atom of glucose and the F atom of MIL-101 (**b**) Mean-squared displacements of glucose in glucose/water/MIL-101 system in the absence and presence of [C_2_mim][Ac].

Figure 6a shows the *g*(*r*) between glucose and the F atom of MIL-101 in glucose/water/MIL-101 system. In the absence and presence of [C_2_mim][Ac], the peak position is identical. However, the peak height drops with IL in the system due to the reduction in adsorption capacity. This reduction can also be evidenced by the H-bonding. As listed in Table 1, the number of H-bonds formed between glucose and MIL-101 is 58.3 in the absence of IL, but reduced to 40.9 in the presence of IL. Furthermore, as shown in Fig. 6b, the mobility of glucose in MIL-101 is reduced in the presence of IL. Since IL is co-adsorbed into MIL-101, the free volume available for glucose to move is less and results in lower mobility. Thus, the presence of IL in aqueous solution has an adverse effect on glucose recovery in MIL-101. In a recent experimental study, however, Prausnitz and coworkers found that glucose uptake in zeolites was increased by [C_2_mim][Ac]^17^. The reason for such a promoting effect was because no IL was adsorbed into microporous zeolites, unlike the situation here, IL is adsorbed into mesoporous MIL-101. Consequently, in their study, the interactions between glucose and water were weakened by IL and glucose was more preferentially adsorbed in zeolites.

**Figure 7:**
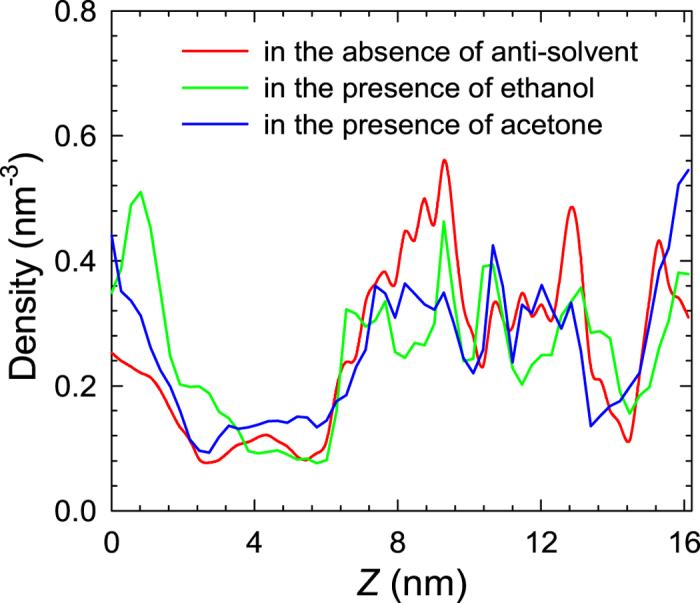
Density profiles of glucose in glucose/water/MIL-101 system in the absence and presence of anti-solvent (ethanol or acetone).

To evaluate the effect of anti-solvent on glucose recovery, Fig. 7 shows the density profiles of glucose in glucose/water/MIL-101 system in the absence and presence of anti-solvent (ethanol or acetone). When anti-solvent is present, the density located between 7 and 16 nm decreases; the average number of adsorbed glucose molecules in MIL-101 is approximately 200, lower than the situation (241.8) in the absence of anti-solvent. Similar to the effect of IL, the adsorption capacity of glucose is also reduced upon adding anti-solvent and the separation factor drops from 2.15 to 1.21. Figure S5 demonstrates that anti-solvent is co-adsorbed into MIL-101 and competes with glucose for adsorption. Therefore, the adsorption capacity of glucose is reduced. Specifically, the number of H-bonds between glucose and MIL-101 is reduced from 58.3 to 47.9 and 37.3 in the presence of ethanol and acetone, respectively.

In summary, we have simulated glucose recovery from aqueous solutions by adsorption in MIL-101. From the analysis of radial distribution functions, the F atom in MIL-101 is identified to be the most favorable site for glucose adsorption and it forms H-bonding with glucose. Upon framework functionalization by −NH_2_ or −CH_3_ group, the adsorption capacity of glucose in MIL-101 decreases due to reduced free volume. Based on the mean-squared displacements, the mobility of glucose also decreases in functionalized MIL-101. If an impurity such as [C_2_mim][Ac] or anti-solvent (ethanol or acetone) is present, the impurity will be co-adsorbed into MIL-101; consequently, the adsorption capacity of glucose and the number of H-bonds between glucose and MIL-101 are reduced. This study provides microscopic insights into the structural and dynamic properties of glucose in MIL-101, and reveals that an impurity has an adverse effect on glucose recovery.

## Methods

Following our previous studies^27^,^30^,^31^, the crystalline structure of MIL-101 was constructed by combining experimental crystallographic data and energy minimization. Each Cr_3_O trimer contained one F atom and the number ratio of F/Cr was 1:3, as experimentally reported^23^. The functionalized MIL-101-X was constructed via replacing an H atom in each phenyl ring by a functional group X (–NH_2_ or –CH_3_). After adding functional groups, the whole unit cell of MIL-101-X was energy minimized using *Forcite* module in Materials Studio^32^. To estimate the atomic charges of MIL-101-X, density functional theory (DFT) calculations were performed on Cr_3_O trimers (see Figure S6). The DFT calculations were conducted by Materials Studio using the Becke exchange plus Lee-Yang-Parr (B3LYP) correlation functional along with the double-*ξ* numerical polarization basis set. From the calculated electrostatic potentials, the atomic charges were fitted using the Merz-Kollman scheme. The dispersion interactions of framework atoms were modeled using the universal force field (UFF)^33^. A number of simulation studies have shown that UFF can accurately predict gas adsorption and diffusion in various MOFs^34^,^35^,^36^.

Figure S7 illustrates the atomic structures of glucose, water, cation and anion of [C_2_mim][Ac], ethanol and acetone. Water was represented by the three-point transferable interaction potential (TIP3P) model, which fairly well mimics the potential energy and pressure of water. The atomic charges of glucose, [C_2_mim]^+^, [Ac]^−^, ethanol and acetone were calculated from the B3LYP method using Gaussian 03 package^37^. Initially, each molecule (or ion) was optimized at 6–311 + G(d,p) basis set and the electrostatic potentials were calculated at 6–311 + + G(d,p) basis set. Thereafter, the atomic charges were determined by the restricted electrostatic potential method, as listed in Table S1. The nonbonded interactions were mimicked by Lennard-Jones (LJ) and Coulombic potentials


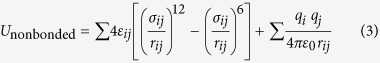


where *ε*_*ij*_ and *σ*_*ij*_ are the well depth and collision diameter, *r*_*ij*_ is the distance between atoms *i* and *j*, *q*_*i*_ is the atomic charge of atom *i*, and *ε*_*0*_ = 8.8542 × 10^−12^ C^2^N^−1^m^−2^ is the permittivity of vacuum. The bonded interactions were described by


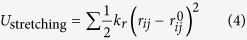



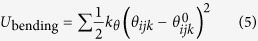



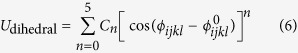


where 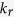, 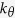 and *C*_*n*_ are the force constants; 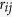, 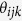 and 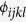 are bond lengths, angles and dihedrals, respectively; 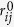, 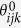 and 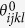 are the equilibrium values. The LJ and bonded potential parameters were adopted from the AMBER force field^38^. As shown in the SI, the predicted densities of [C_2_mim][Ac], ethanol and acetone match well with experimental data, suggesting that the force field parameters are accurate.

As listed in Table S2, six simulation systems were considered in this study for glucose recovery. In system 1, MIL-101 was examined for the adsorption of glucose from aqueous solution. Figure S8 illustrates the initial simulation cell of system 1. The cell dimensions along the *x* and *y*-axis were equal to the unit cell length of MIL-101 (88.87 Å), while the cell dimension along the *z*-axis was 164 Å. In systems 2–3, the MIL-101-NH_2_ and MIL-101-CH_3_ were used. Moreover, the effects of 5 wt% of [C_2_mim][Ac] and 10 wt% of anti-solvents (ethanol and acetone) were explored separately in systems 4–6. The wt% was estimated with respect to solution phase (water and glucose). For each system, the number of glucose molecules was 330, representing 20 wt% of glucose in solution. Firstly, the steepest descent method was used for energy minimization with a maximum step size of 0.01 nm and a force tolerance of 10 kJ mol^−1^nm^−1^. Then, non-equilibrium molecular dynamics (MD) simulation was performed at 298 K using Gromacs v.4.5.3^39^. The temperature was controlled by velocity-rescaled Berendsen thermostat with a relaxation time of 0.1 ps. MIL-101-X framework was assumed to be rigid during simulation with the framework atoms fixed. A cutoff of 1.4 nm was used to evaluate the LJ interactions, and the electrostatic interactions were calculated using the particle-mesh Ewald method with a grid spacing of 0.12 nm and a fourth-order interpolation. The equations of motion were integrated with a time step of 0.5 fs by leapfrog algorithm. To mimic a stirring effect usually present in experimental test, an external acceleration *a*_ext_ = 0.01 nm/ps^2^ was exerted on solvent (including water, [C_2_mim][Ac], ethanol or acetone). The simulation duration was 35 ns and the last 1 ns trajectory was used for ensemble averages. From the independent runs with different initial configurations, as shown in Figure S9, the number of glucose adsorbed at equilibrium was found to be close.

## Additional Information

**How to cite this article**: Gupta, K. M. *et al*. Glucose recovery from aqueous solutions by adsorption in metal-organic framework MIL-101: a molecular simulation study. *Sci. Rep*. **5**, 12821; doi: 10.1038/srep12821 (2015).

## Supplementary Material

Supplementary Information

Supplementary Video S1

## Figures and Tables

**Figure 1:**
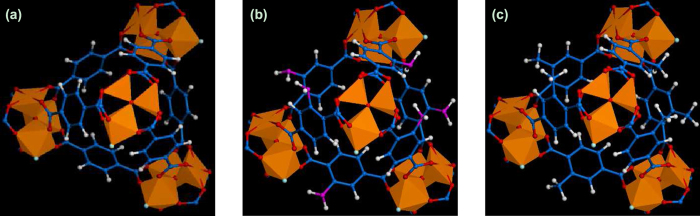
Supertetrahedra in (**a**) MIL-101 (**b**) MIL-101-NH_2_ (**c**) MIL-101-CH_3_. Cr_3_O clusters are denoted as orange polyhedral, C: blue, O: red, F: cyan, N: pink, H: white.

**Table 1:** Number of H-bonds between glucose and MIL-101.

System	H-bonds
Glucose/water/MIL-101	58.3
Glucose/water/IL/MIL-101	40.9
Glucose/water/ethanol/MIL-101	47.9
Glucose/water/acetone/MIL-101	37.3
