# Supplementary material for: Glucose recovery from aqueous solutions by adsorption in metal–organic framework MIL-101: a molecular simulation study
Source: Sci Rep. 2015 Aug 5;5:12821. doi: 10.1038/srep12821 (PMC4525290; doi:10.1038/srep12821)
Supplement: Supplementary Information [file srep12821-s1.pdf]

Supplementary Information

Glucose recovery from aqueous solutions by adsorption in metal–organic framework MIL-101: a molecular simulation study

Krishna M. Gupta, Kang Zhang and Jianwen Jiang*

Department of Chemical and Biomolecular Engineering, National University of Singapore, 117576, Singapore

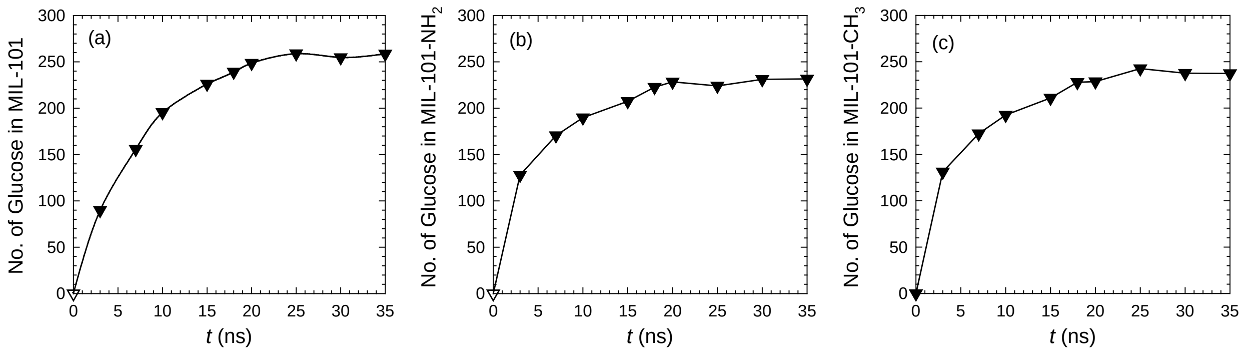

Figure S1 | Numbers of glucose adsorbed in (a) MIL-101 (b) MIL-101-NH_2 and (c) MIL-101-CH_3 versus simulation time.

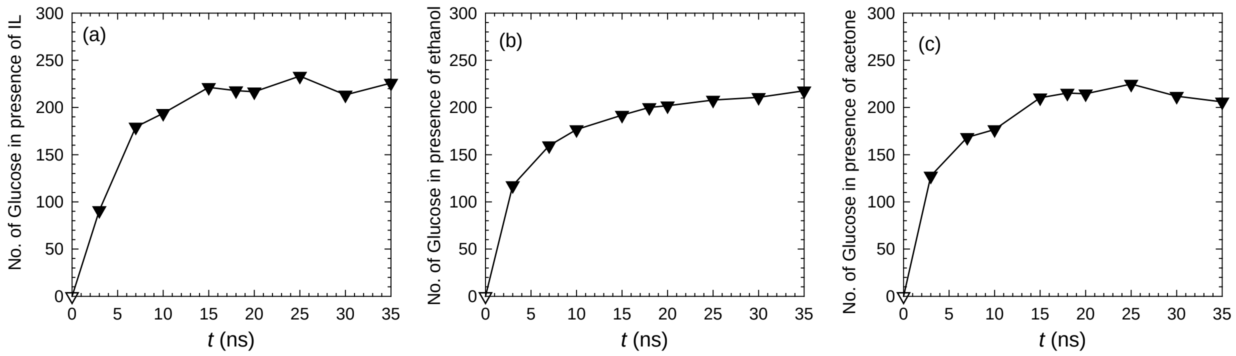

Figure S2 | Numbers of glucose adsorbed in MIL-101 in the presence of (a) IL (b) ethanol and (c) acetone versus simulation time.

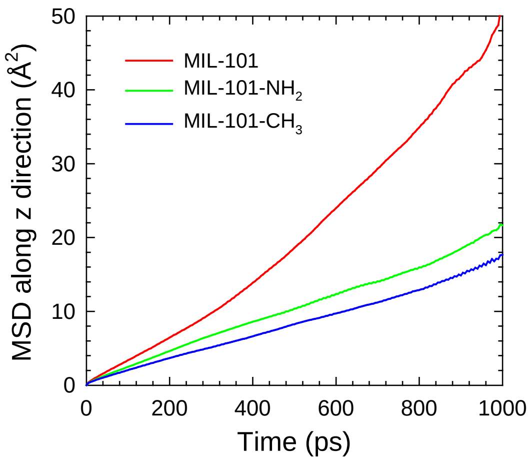

Figure S3 | Mean-squared displacements of glucose along the z-axis in glucose/water/MIL-101-X systems.

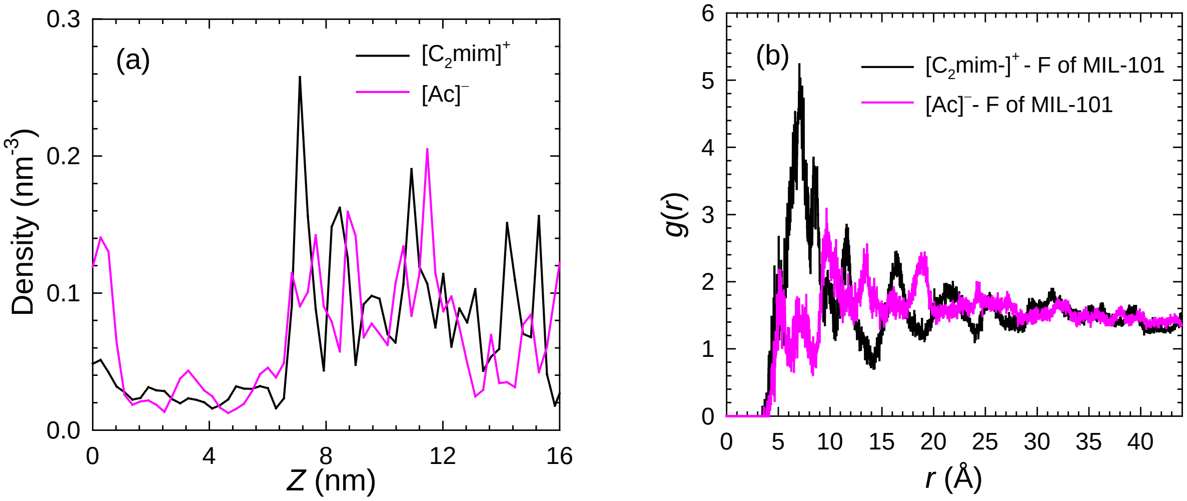

Figure S4 | (a) Density profiles of $[C_2mim]^+/[Ac]^-$ and (b) radial distribution functions between $[C_2mim]^+/[Ac]^-$ and the F atom of MIL-101 in glucose/water/MIL-101 system in the presence of $[C_2mim][Ac]$.

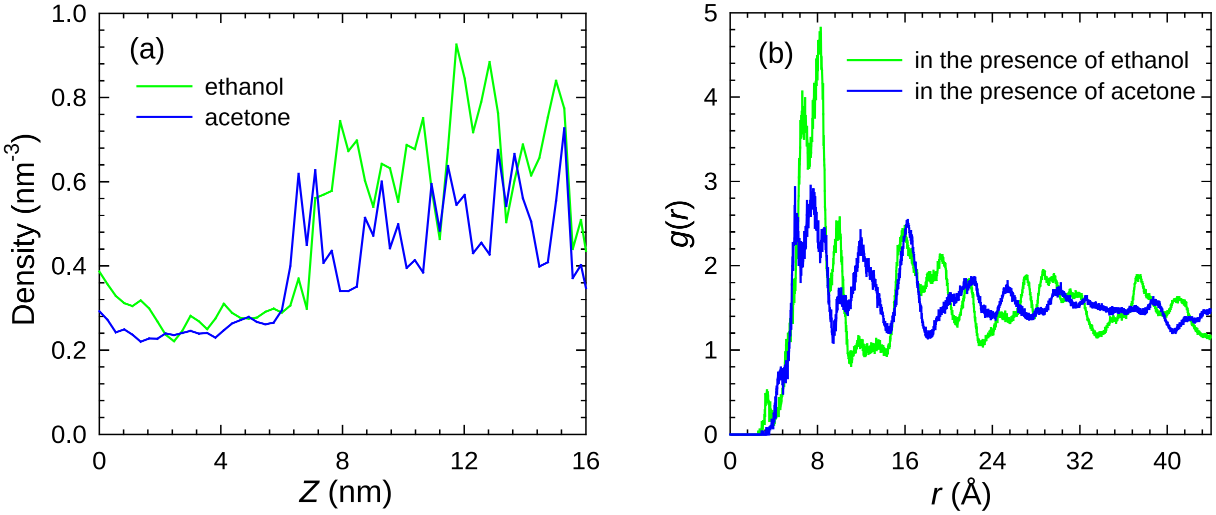

Figure S5 | (a) Density profiles of anti-solvents and (b) radial distribution functions between anti-solvent and the F atom of MIL-101 in glucose/water/MIL-101 system in the presence of anti-solvent (ethanol or acetone).

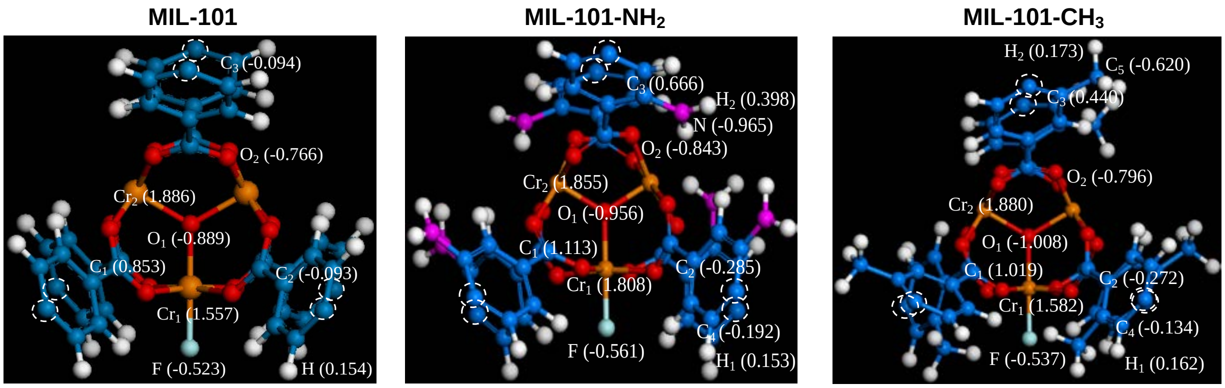

Figure S6 | Atomic charges of Cr_3O trimer in MIL-101, MIL-101-NH_2 and MIL-101-CH_3. The cleaved bonds indicated by circles were saturated by methyl groups.

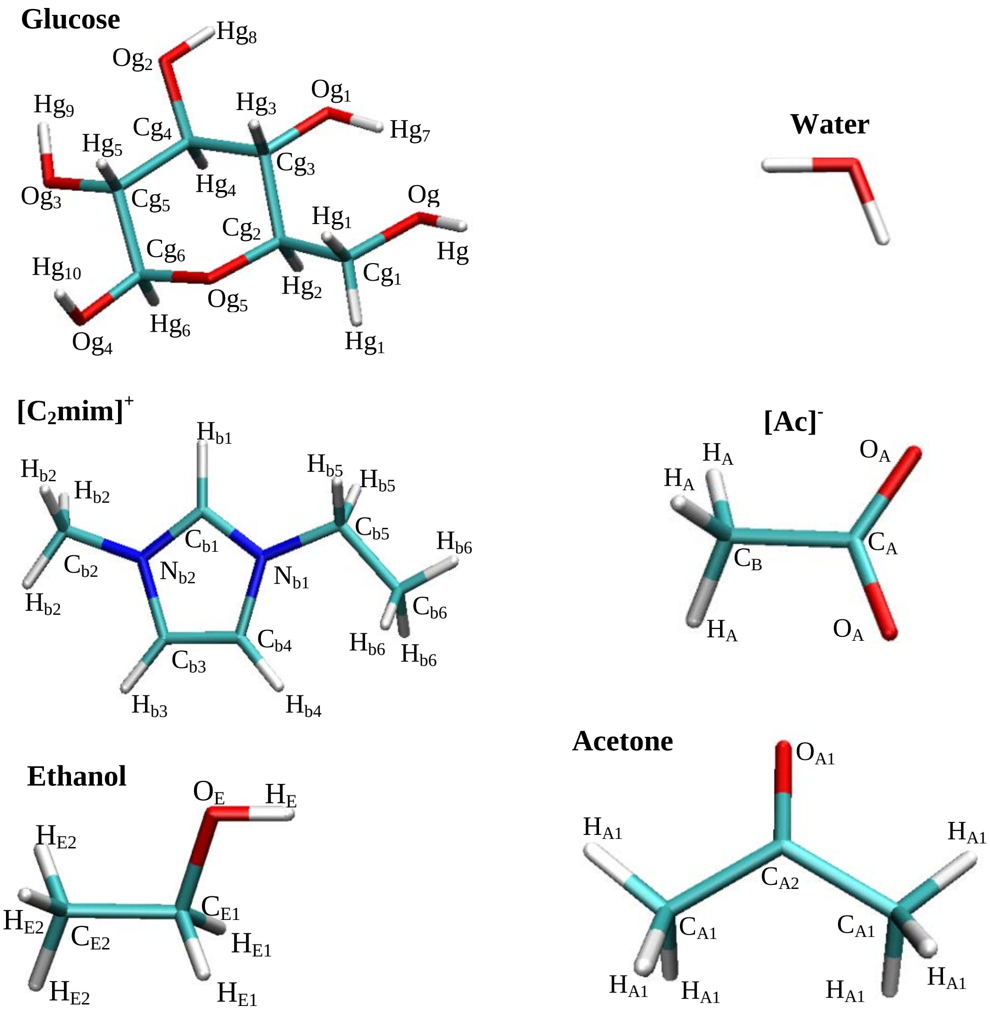

Figure S7 | Atomic structures of glucose, water, $[C_2mim]^+$, $[Ac]^-$, ethanol and acetone.

Table S1. Atomic charges of glucose, $[C_2mim]^+$, $[Ac]^-$, ethanol and acetone.

Glucose

Atom	C_{g1}	C_{g2}	C_{g3}	C_{g4}	C_{g5}	C_{g6}	O_g	O_{g1}	O_{g2}	O_{g3}
**Charge**	0.229	0.328	0.065	0.258	0.022	0.728	-0.714	-0.678	-0.688	-0.691
**Atom**	O_{g4}	O_{g5}	H_g	H_{g1}	H_{g2}	H_{g3}	H_{g4}	H_{g5}	H_{g6}	H_{g7}
**Charge**	-0.641	-0.683	0.431	0.027	0.016	0.070	0.027	0.083	0.017	0.448
**Atom**	H_{g8}	H_{g9}	H_{g10}							
**Charge**	0.454	0.465	0.404							

$[C_2mim]^+$

Atom	C_{b1}	C_{b2}	C_{b3}	C_{b4}	C_{b5}	C_{b6}	N_{b1}	N_{b2}	H_{b1}	H_{b2}
**Charge**	-0.157	-0.351	-0.287	-0.113	-0.078	-0.227	0.143	0.291	0.217	0.173
**Atom**	H_{b3}	H_{b4}	H_{b5}	H_{b6}						
**Charge**	0.258	0.218	0.127	0.104						

$[Ac]^-$

Atom	C_A	C_B	O_A	H_A
**Charge**	1.026	-0.338	-0.884	0.026

Ethanol

Atom	C_{E1}	C_{E2}	O_E	H_E	H_{E1}	H_{E2}
**Charge**	0.543	-0.276	-0.725	0.416	-0.087	0.072

Acetone

Atom	C_{A1}	C_{A2}	O_{A1}	H_{A1}
**Charge**	-0.481	0.779	-0.566	0.125

To validate the force field parameters used for $[C_2mim][Ac]$, ethanol and acetone, MD simulations were performed. For $[C_2mim][Ac]$, the system was annealed from 500 to 350 K with a temperature interval of 50 K, and MD simulation was run for 2 ns at each temperature; finally, the system was equilibrated at 323 K and 1 bar for 20 ns. The density of $[C_2mim][Ac]$ was predicted to be 1.105 g/cm^3, in good accord with experimental value (1.086 g/cm^3).[1] For ethanol and acetone, the systems were run at 298 K and 1 bar for 20 ns. The densities were estimated to be 808 kg/m^3 and 787 kg/m^3, close to experimentally measured values (789 and 791 kg/m^3).[2,3]

1. D. T. Bowron, C. D'Agostino, L. F. Gladden, C. Hardacre, J. D. Holbrey, M. C. Lagunas, J. McGregor, M. D. Mantle, C. L. Mullan and T. G. A. Youngs, *J. Phys. Chem. B*, 2010, **114**, 7760.
2. P. J. Berryman, D. A. Faux and D. J. Dunstan, *Phys. Rev. B*, 2007, **76**, 104303.
3. http://www.chemicalbook.com/ChemicalProductProperty_EN_CB3130928.htm

Table S2. Six simulation systems.

System	MOF	No. of $[C_2mim][Ac]$	No. of ethanol	No. of acetone
1	MIL-101	-	-	-
2	MIL-101-NH_2	-	-	-
3	MIL-101-CH_3	-	-	-
4	MIL-101	88 (5 wt%)	-	-
5	MIL-101	-	646 (10 wt%)	-
6	MIL-101	-	-	512 (10 wt%)

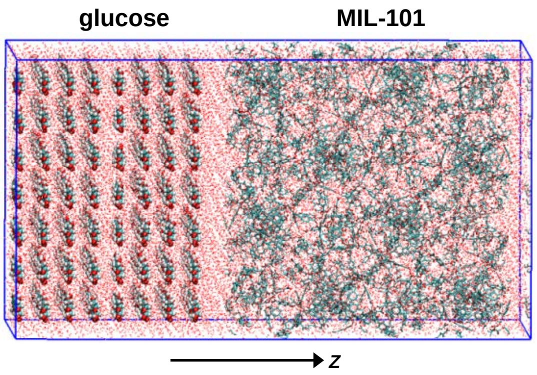

Figure S8 | Initial simulation cell of glucose/water/MIL-101 system. Water molecules are in red.

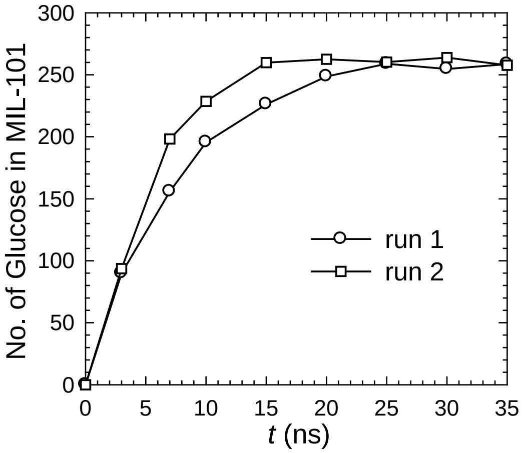

Figure S9 | Numbers of glucose adsorbed in MIL-101 from two independent runs.
